# β-Cyclodextrin/Thymol Microcapsule-Embedded Starch Coatings for Synchronized Antimicrobial Release and Shelf-Life Extension in Blueberries

**DOI:** 10.3390/foods14173132

**Published:** 2025-09-07

**Authors:** Xiangyue Li, Yuxin Liu, Jiayi Zheng, Xiaoyi Zhu, Weirui Fang, Shanshan Lei, Weiran Zhuang, Jing Wu, Tong Hao, Sulin You, Xi Wei, Wen Qin, Yaowen Liu, Mingrui Chen

**Affiliations:** College of Food Science, Sichuan Agricultural University, Ya’an 625014, China; 18782138289@163.com (X.L.);

**Keywords:** thymol, β-cyclodextrin, microencapsulation, coating, blueberries

## Abstract

An eco-friendly composite coating was developed for blueberry preservation through the incorporation of thymol-loaded β-cyclodextrin microcapsules (THY@β-CD) into a potato starch (PO) matrix. Microencapsulation at an optimal wall-to-core ratio of 13:1 achieved a THY encapsulation efficiency of 73.24%. Structural analyses confirmed the successful formation of an inclusion complex, which enhanced thermal stability and provided a controlled release profile governed by Fickian diffusion mechanisms. When applied to blueberries, the coating significantly reduced weight loss by 22%, delayed softening, and more effectively preserved anthocyanin content compared to uncoated fruit during 10-day storage. Furthermore, it well-maintained the sensory quality and visual appeal of the fruit. These results demonstrate that the THY@β-CD/PO coating synergistically integrates sustained antimicrobial delivery with matrix compatibility, offering a promising natural alternative to synthetic preservatives for extending the shelf life of blueberries.

## 1. Introduction

Blueberries (*Vaccinium* spp.), members of the Ericaceae family, represent a globally significant berry crop valued for their high concentration of bioactive compounds and underlying documented health benefits. Their key phytochemical constituents comprise anthocyanins (primary pigments including malvidin, delphinidin, and cyanidin glycosides), flavonols (e.g., quercetin, myricetin, and kaempferol derivatives), flavan-3-ols (catechins and proanthocyanidins), and phenolic acids (e.g., chlorogenic and caffeic acids) [1]. These compounds exhibit potent antioxidant and anti-inflammatory activities, contributing to diverse health-promoting effects [2], while their characteristic sweet–sour profile enhances consumer preference. Postharvest quality deterioration primarily stems from respiratory senescence, mechanical damage due to epidermal fragility [3], and microbial proliferation accelerating decay. Conventional preservation methodologies include refrigeration (reducing weight loss, maintaining firmness and acidity, typically requiring complementary approaches) [4], hot-air treatments (enhancing disease resistance, yet potentially compromising fruit quality and energy efficiency) [5], controlled atmosphere storage (regulating metabolic rates through gas composition modulation, albeit requiring precise gas ratios with limited efficacy against specific spoilage microorganisms), and irradiation (reducing microbial loads while preserving quality, notwithstanding risks of accelerated senescence and peel damage at improper dosages) [6,7,8]. Chemical preservatives such as methyl jasmonate, 1-methylcyclopropene, salicylic acid, and SO_2_ applied via fumigation/coating face limitations including residue concerns, potential toxicity, and transient efficacy [9]. Consequently, plant essential oils with broad-spectrum antimicrobial activity and environmental compatibility have gained prominence [10]. Among these, thymol (THY) demonstrates significant preservation potential [11], although its direct incorporation into polymer coatings (e.g., chitosan, gelatin, starch) [12,13,14] is constrained by (1) limited aqueous solubility causing heterogeneous dispersion, migration, and compromised coating integrity, and (2) high volatility, leading to rapid active component loss and pungent odor impacting sensory attributes [15]. To circumvent these limitations and enhance THY stability, aqueous dispersibility, controlled release, and odor mitigation within coating systems, pre-encapsulation prior to matrix integration has emerged as a critical research strategy [16].

Embedding technology effectively mitigates these limitations by encapsulating bioactive molecules within macromolecular carrier systems. β-Cyclodextrin (β-CD), possessing a hydrophobic cavity, forms stable inclusion complexes with non-polar guests via non-covalent interactions. This significantly enhances THY aqueous solubility, masks undesirable flavors, and improves thermal stability [17], providing a critical pathway for food-grade applications. Notably, β-CD has been utilized as a cryoprotectant to stabilize THY-essential-oil solid liposomes [18]. Recent advances have further explored the application of β-CD inclusion complexes in sustainable antimicrobial packaging, demonstrating their efficacy in extending the shelf-life of various fruits [19,20]. Among polysaccharides, potato starch (PO) is widely incorporated into edible coatings due to its high amylopectin content, superior oxygen barrier properties, and mechanical strength [21]. Zhang et al. [22] demonstrated that nano-SiO_2_/PO composite coatings exhibit enhanced water resistance, mechanical properties, UV shielding, and thermal resistance. However, the inherent antimicrobial efficacy of starch-based coatings remains limited, necessitating integration with supplementary antimicrobial agents to extend food shelf-life [23].

Current research on β-CD-microencapsulated THY within starch-based composite coatings for blueberry preservation is limited. In this study, THY was encapsulated in β-CD to form inclusion complexes (THY@β-CD), subsequently incorporated into a PO matrix to fabricate active composite coatings. The physical properties (hygroscopicity, wettability) and antimicrobial efficacy of THY@β-CD/PO coatings were systematically characterized. The optimized coating formulation was then evaluated for postharvest quality preservation in blueberries under controlled storage conditions. Key parameters assessed included weight loss, firmness, decay incidence, and anthocyanin content. This natural coating system demonstrates significant potential for blueberry preservation, contributing to the development of safe and effective strategies to extend shelf-life, maintain nutritional quality, and reduce postharvest losses.

## 2. Materials and Methods

### 2.1. Materials

All chemical reagents, including anhydrous ethanol, hydrochloric acid (HCl), potassium iodate (KIO_3_), sodium hydroxide (NaOH), methanol, potato dextrose agar medium (PDA), potassium iodide (KI), and phenolphthalein, were of analytical grade. Food-grade materials, namely THY, β-CD, glycerol, and PO, were procured from Shanghai Zhan Yi Bakery Co., Shanghai, China. Fungal strains (*Alternaria alternate*, *Penicillium* sp., and *Aspergillus niger*) were provided by the Key Laboratory of Agro-Products Processing and Storage at Sichuan Agricultural University, China.

Fresh blueberries (*Vaccinium corymbosum* ‘Baldwin-T-117’, a late-ripening cultivar) were harvested from Guihe Agricultural Park (Qingbaijiang District, Chengdu, China). The mean mass of individual berries was 1.0 ± 0.5 g. All fruits met the maturity criteria specified in Chinese agricultural standard NY/T 3033-2016 [24].

Post-harvest handling was performed under optimized protocols. Berries were transported to the laboratory within 2 h under temperature control (4–6 °C) and pre-cooled at 4 °C for 4 h. Surface sterilization was conducted by immersion in 200 ppm sodium hypochlorite for 2 min, followed by triple rinsing with distilled water (1:3, *w*/*v*). Residual moisture was removed by air-drying under ambient conditions (25 °C, 55% RH) until no visible surface moisture remained (approximately 20 min) before further treatments.

### 2.2. Preparation of THY@β-CD Microcapsules

Microcapsules were prepared using a modified saturated aqueous solution method [25]. Specifically, β-CD was dissolved in deionized water at a mass-to-volume ratio of 1:15 under stirring at 70 °C and 600× *g* for 20 min to form a homogeneous wall-material solution. Simultaneously, THY was dissolved in anhydrous ethanol at a mass-to-volume ratio of 1:2 under ambient stirring conditions (600× *g*). The THY solution was added dropwise to the β-CD solution under continuous magnetic stirring (HJ-6A magnetic stirrer, Guohua Electric Appliance Co., Ltd., Changzhou, China) at 50 °C and 600× *g* to achieve wall-to-core mass ratios ranging from 11:1 to 15:1 (β-CD: THY). After stirring for 2 h, the mixture was refrigerated at 4 °C for 12 h. The precipitate was then vacuum-filtered and repeatedly washed with anhydrous ethanol (2–3 cycles) to remove uncomplexed THY. Finally, the product was vacuum-dried (DHG- series vacuum drying oven, Dongxing Instrument Co., Ltd., Shanghai, China) at 25 °C for 12 h until constant mass was attained. The resulting microcapsules were stored in amber vials at 4 °C for subsequent analysis.

### 2.3. Encapsulation Efficiency Measurement

A standard THY solution (5 mg/mL) was prepared by dissolving 500 mg THY in ethanol with volumetric dilution to 100 m. Aliquots (0.1, 0.2, 0.3, 0.4, 0.5 mL) were transferred to 10 mL volumetric flasks for absorbance measurement at 244/274 nm (UV-4802S UV-Vis spectrophotometer, Unico Instrument Co., Ltd., Shanghai, China), establishing a standard curve from differential absorbance (A, Equation (1)). For total THY quantification, 0.5 g THY@β-CD was dissolved in 22.5 mL anhydrous ethanol, ultrasonicated (KQ5200E ultrasonic cleaner, Unico Instrument, China; 50 °C, 15 min), and centrifuged (10,732× *g*, 15 min), with supernatant absorbance measured at 244/274 nm. Unencapsulated THY was determined by washing 0.5 g THY@β-CD with 22.5 mL anhydrous ethanol (3 cycles) under vacuum filtration, measuring combined filtrate absorbance. Encapsulated THY analysis involved dissolving the washed microcapsules in ethanol, repeating ultrasonication/centrifugation, and measuring supernatant absorbance. THY contents were calculated via the standard curve (triplicates), with encapsulation efficiency (EE) derived from Equation (2):

A = A_274_ − A_244_ (1)

EE (%) = (A − B)/A × 100%(2)

where A_274_ and A_244_ represent the absorbance of THY at wavelengths of 274 nm and 244 nm, respectively; A, B, are the total THY content and unembedded THY content, respectively.

### 2.4. Characterization of THY@β-CD Microcapsules

Morphological analysis was conducted by scanning electron microscopy (JSM-6610LV SEM, JEOL Ltd., Tokyo, Japan) at 1 kV following gold sputter coating. Particle size distribution and polydispersity index (PDI) were determined via dynamic light scattering (Zetasizer Nano ZS particle size analyzer, Malvern Panalytical Ltd., Malvern, UK) using 200-fold aqueous dilutions, with both parameters derived from the Stokes-Einstein equation based on triplicate measurements. X-ray diffraction (XRD) patterns were acquired on a Bruker D8 Advance X-ray diffractometer (Bruker AXS GmbH, Karlsruhe, Germany) using Cu Kα radiation (λ = 0.15418 nm) with a Ni filter, operating at 40 kV and 40 mA. Data were collected over a 2θ range of 5–80° at a scan rate of 0.2°/min. Thermal properties were assessed by differential scanning calorimetry (Q200 modulated DSC, TA Instruments, New Castle, DE, USA) using 2–4 mg samples sealed in aluminum crucibles under N_2_ purge (30 mL/min), with temperature ramped from 40 to 400 °C at 10 °C/min against an empty crucible reference.

### 2.5. Release Behavior of THY@β-CD Microcapsules

Microcapsules demonstrating the highest EE (per Section 2.3) and a physical mixture of THY and β-CD at an identical β-CD: THY mass ratio (non-encapsulated control) were stored under dark conditions at 26 °C ± 1 °C and 4 °C ± 0.5 °C. At each interval, homogeneous solid aliquots (1.0 ± 0.02 g) of the microcapsules were collected. The retained THY was quantitatively extracted into anhydrous ethanol and analyzed spectrophotometrically as per Section 2.3, ensuring that all measurements were performed against the standard curve in an identical solvent matrix to preclude systematic error. The core retention rate (CR, %) was calculated as Equation (3):

Core Retention Rate (%) = [1 − (E_0_ − E_n_)/E_0_] × 100% (3)

where E_0_ is encapsulation efficiency at day 0; E_n_ is encapsulation efficiency at day n.

The release profiles were subsequently fitted to three established kinetic models (first-order, Higuchi, and Korsmeyer–Peppas) (Table 1) via nonlinear regression using the Levenberg–Marquardt algorithm in OriginPro 2022. Model selection was based primarily on the Akaike Information Criterion (AIC) and the root mean square error (RMSE), with the model exhibiting the lowest AIC and RMSE values being selected.

### 2.6. Preparation of Composite Coatings

A starch-based coating matrix was prepared by dispersing 4 g PO in 100 mL distilled water within a 250 mL borosilicate beaker. Homogenization was achieved via mechanical stirring (300× *g*; RW-20 digital overhead stirrer, IKA, Staufen, Germany). Gelatinization proceeded in a precision water bath (SW23 water bath, Julabo, Seelbach, Germany, 90 ± 0.5 °C, 30 min) under continuous agitation (150× *g*). The matrix was plasticized with 1.5 g glycerol and equilibrated at 40 °C for 20 min. Five microcapsule-loaded formulations were prepared by incorporating 2.5–12.5 wt% THY@β-CD microcapsules into the gelatinized matrix under magnetic stirring (150× *g*) until complete dissolution (15–20 min). Homogeneity was maintained during storage at 50× *g*. Formulations were designated as THY@β-CD/PO-X (X = 2.5, 5.0, 7.5, 10.0, 12.5) and stored in amber vials at 25 ± 1 °C prior to casting.

### 2.7. Characterization of Composite Coatings

#### 2.7.1. Scanning Electron Microscopy (SEM)

Coating solutions were cast into PTFE molds (150 × 150 × 10 mm), cured at 30 °C/50% RH for 48 h, and peeled for analysis. Surface/cross-section morphology was examined by SEM (JSM-6610LV scanning electron microscope, JEOL Ltd., Tokyo, Japan) at 5 kV after gold sputtering.

#### 2.7.2. Fourier Transform Infrared Spectroscopy

The molecular interactions of the samples were analyzed using FTIR [26]. Vacuum-dried samples (THY, β-CD, THY@β-CD, PO and composite coatings) were mixed with KBr (1:50 *w*/*w*), finely ground, and pressed into pellets. FTIR spectra (Tensor II FTIR spectrometer, Bruker Optics GmbH & Co. KG, Bremen, Germany) were acquired from 4000 to 650 cm^−1^ (4 cm^−1^ resolution) against KBr background.

#### 2.7.3. Stereomicroscopic Characterization of Composite Coatings

Blueberries coated with THY@β-CD/PO-X (X = 2.5, 5, 7.5, 10, 12.5 wt%; *n* = 3/group) were sectioned equatorially (1–2 mm) with microtome blades. Coating morphology and microcapsule distribution were analyzed under stereomicroscope (Stemi 508 stereo microscope, Carl Zeiss Microscopy GmbH, Jena, Germany) at 10–40× magnification (3 random fields/sample).

#### 2.7.4. Determination of Hygroscopicity of Composite Coatings

THY@β-CD microcapsule coatings were cut into circular specimens (80 mm diameter) with uniform thickness and no surface imperfections. Samples were conditioned at ambient temperature (20–26 °C) and relative humidity (54–70%). Mass changes were monitored gravimetrically for 10 consecutive days. Moisture absorption was quantified as the daily mass gain percentage relative to the initial mass (day 0). Triplicate measurements per coating specimen were conducted with mean values reported.

#### 2.7.5. Wettability of Composite Coatings

Coating solutions (0.2 mm thickness) were cast and cured (25 °C, 24 h). Static contact angles were measured (OCA 25 contact angle goniometer, DataPhysics Instruments GmbH, Filderstadt, Germany) by dispensing 3 μL ultrapure water onto surfaces at 25 °C. Images captured within 10 s (3 locations/sample).

#### 2.7.6. Determination of the Viscosity of Composite Coatings

Viscosity was determined using a rotational rheometer (MCR 302 rheometer, Anton Paar GmbH, Graz, Austria) with parallel plates (50 mm, 1 mm gap). Samples (2 mL) were equilibrated at 25 °C, pre-sheared (60 s), and scanned from 1 to 100 s^−1^ in steady-shear mode (triplicates).

### 2.8. Determination of the Antimicrobial Performance of Composite Coatings

The antifungal activity of the composite coating solutions was assessed against *Alternaria alternate*, *Aspergillus niger*, and *Penicillium* sp. according to a previously reported method [27] with modifications. Comparative agar diffusion assays were conducted using THY@β-CD-10 (10 wt% microcapsules), unencapsulated THY suspended in 0.1% Tween 80 aqueous solution, and pure β-CD, with all formulations adjusted to equivalent active compound concentrations. Aliquots (200 μL) of each coating solution at concentrations ranging from 2.5 to 12.5 wt% were applied to the surface of PDA plates and spread uniformly with a sterile L-shaped glass spreader. Fungal strains were initially revitalized on PDA slants for 48 h at their optimal growth temperatures (25–30 °C). Mycelial disks (5 mm in diameter), obtained from the actively growing margins of colonies, were aseptically transferred onto the pre-coated PDA plates. Following inoculation, the plates were incubated inverted in darkness at species-specific temperatures for 5 days. Antifungal efficacy was determined by measuring colony diameters using the cross method and calculating percentage inhibition relative to untreated controls.

For morphological examination, fungal samples exhibiting growth inhibition after exposure to the composite coatings were prepared according to the procedures detailed in Section 2.7.3 and characterized using SEM to assess ultrastructural damage to hyphae and spores.

### 2.9. Postharvest Treatment of Blueberries with Composite Coatings

Pretreated blueberries were allocated to three groups: (1) the composite coating treatment group immersed in experimental solution (1 min) with subsequent drainage of excess liquid and air-drying at 25 ± 1 °C; (2) the PO coating control group subjected to identical immersion and drying procedures; and (3) the untreated blank group air-dried directly. All samples were stored in aerated polypropylene (PP) preservation boxes at 20–26 °C and 54–70% relative humidity, with analytical sampling performed at 48 h intervals.

### 2.10. Characterization of Storage Quality of Post-Harvest Blueberries

#### 2.10.1. Sensory Evaluation

A sensory panel (*n* = 10; 5 males, 5 females) recruited from the College of Food Science, Sichuan Agricultural University evaluated blueberry texture, appearance, and odor daily over a 10-day period. The protocol synthesized established sensory analysis principles [28] with adaptations from Jia et al. [29] and Su et al. [30] utilizing a standardized scoring matrix (Table 2) with quantitative descriptors for each attribute. Panelists independently rated samples against defined criteria. Results were statistically analyzed and expressed as mean ± standard deviation to ensure methodological robustness and mitigate evaluator bias. The study was conducted in accordance with the Declaration of Helsinki, and ap-proved by the Sichuan Agricultural University Animal Ethical and Welfare Committee. (number 20240529). Informed consent for publication was obtained from all identifiable human participants.

#### 2.10.2. Determination of Fruit Peel Appearance and Color Difference

Blueberry epidermal morphology was documented via calibrated macrophotography under D65 standard illumination, with one randomly selected berry per treatment group imaged. Quantitative colorimetry employed a CS-10 colorimeter (Chinspec Technology Co., Ltd., Hangzhou, China) measuring CIELAB coordinates (L, a, b*). For fruit skin analysis, five berries per group were surface-cleaned, with three equidistant equatorial measurements averaged per specimen. Coating color was determined through triplicate measurements against a barium sulfate reference tile. Total color difference (ΔE) was calculated using Equation (4):

(4) $$∆E\;=\;\sqrt{(L\;-\;L_{0}{)}^{2}\;+\;(a\;-\;a_{0}{)}^{2}+(b\;-\;b_{0}{)}^{2}}$$

where *L*, *a* and *b* are the CIELab color values of the sample to be tested, and *L*_0_, *a*_0_ and *b*_0_ are the CIELab color values of the standard whiteboard.

#### 2.10.3. Determination of Fruit Hardness

Fruit hardness was determined using a GY-4 penetrometer (Aidebao Instrument Co., Ltd., Yueqing, China) with ten blueberries per group. Triplicate measurements per fruit were averaged following standardized testing protocols.

#### 2.10.4. Determination of Fruit Decay Rate

Blueberry decay severity was classified using a modified five-tier scale adapted from Jiang et al. [31], defined by the proportional surface area exhibiting visible decay: Grade 0 (0%), Grade 1 (<25%), Grade 2 (25–50%), Grade 3 (50–75%), and Grade 4 (>75%). Based on this scale, the decay rate within each treatment group was calculated as the proportion of decayed berries (Grades 1–4) relative to the total berries assessed, according to Equation (5):

(5) $$Fruit\;Decay\;Rate=\frac{decay\;level\times Number\;of\;samples\;at\;that\;level}{highest\;decay\;level\times total\;number\;of\;samples}\times 100\%$$

#### 2.10.5. Determination of Fruit Weight Loss Rate

The mass loss percentage of blueberry fruits quantifies the percentage reduction in weight relative to initial mass, occurring over a defined storage period under controlled conditions. This parameter was calculated using Equation (6):

Weight Loss Rate = (m_0_ − m_t_)/m_0_ × 100% (6)

where m_0_ is the weight of blueberry fruit before storage; m_t_ is the weight of blueberry fruit after storage.

#### 2.10.6. Anthocyanin Content Determination

Blueberry homogenate (1 g) was combined with 20 mL of 60% ethanol (pH 3.0) and subjected to water bath extraction at 40 °C for 2 h. The supernatant was recovered by centrifugation (10,732× *g*, 15 min). Total anthocyanin content was quantified using pH differential spectrophotometry [32] with modifications. Anthocyanin extract (0.5 mL) was added to 4.5 mL of KCl-HCl buffer (pH 1.0) and 4.5 mL of NaOAc-HOAc buffer (pH 4.5). Corresponding blanks contained 0.5 mL of 1% HCl-methanol in each buffer. Absorbance was measured at 510 nm and 700 nm, with total anthocyanin content calculated employing Equations (7) and (8):

ΔA = (A_510nm_ − A_700nm_) pH_1.0_ − (A_510nm_ − A_700nm_) pH_4.5_ (7)

Anthocyanin Content (mg/g) = ∆A × M × V × F/Ɛ × m(8)

where M (449.2 g/mol) is the relative molecular weight of cyanidin-3-glucoside; m (g) is the mass of the sample; V (mL) is the volume of the extraction solvent; A is the absorbance difference between pH_1.0_ and pH_4.5_ buffers at 520 nm and 700 nm; F is the dilution factor of the extract; Ɛ (26,900 L·mol^−1^·cm^−1^) is the molar absorption coefficient of cyanidin-3-glucoside.

### 2.11. Statistical Analysis

Statistical analyses were conducted using SPSS 22.0 (IBM Corp., Armonk, NY, USA). Results were reported as mean ± standard deviation (SD). Analyses for Section 2.5 employed repeated-measures two-way ANOVA with Bonferroni adjustment to assess treatment-by-temperature interactions at a significance level of *p* < 0.05. Data in Section 2.8, Section 2.10.3, Section 2.10.4, Section 2.10.5 and Section 2.10.6 were evaluated using two-way ANOVA with Bonferroni post hoc testing. All other datasets were analyzed by one-way ANOVA followed by Tukey’s HSD test for multiple comparisons. Prior to analysis, all models were validated for conformity to assumptions of homogeneity (Levene’s test, *p* > 0.05) and normality (Shapiro–Wilk test, *p* > 0.05). Data visualization was performed using OriginPro 2018 (OriginLab Corp., Northampton, MA, USA).

## 3. Results and Discussions

### 3.1. The Embedding Rate, Particle Size, Zeta and PDI of THY@β-CD Microcapsules Prepared with Different Wall Core Ratio

Table 3 summarizes the key performance parameters of microcapsules prepared at different wall-to-core ratios, which significantly influenced EE, particle size, and zeta potential. EE, determined using a validated standard curve for THY (y = 0.0167x + 0.0048, R^2^ = 0.9998) conforming to the Beer–Lambert law, increased initially from 66.45 ± 1.21% (1:11) to a maximum of 73.24 ± 0.82% at 1:13 (*p* < 0.05), then declined to 66.73 ± 0.65% at 1:15 (*p* < 0.05). This non-monotonic trend aligns with previous reports [33] and is attributed to optimal complexation at intermediate ratios due to sufficient cavity availability, whereas excess wall material induces steric hindrance or self-association, limiting THY incorporation. Particle size exhibited a concave trend, decreasing from 2120.79 ± 32.73 nm (1:11) to 1927.77 ± 25.39 nm (1:13) (*p* < 0.05), then increasing to 2184.34 ± 21.13 nm (1:15) (*p* < 0.05), suggesting improved emulsification followed by aggregation. PDI values (0.68–0.73, *p* > 0.05) indicate broad size distribution, representing a limitation for encapsulation stability and release kinetics, and highlighting the need for improved monodispersity in future formulations [34]. In contrast, the absolute zeta potential increased monotonically from |13.75 ± 0.15| mV to |17.98 ± 0.12| mV (*p* < 0.05), consistent with enhanced surface contribution from the wall material. Based on its superior EE and minimal particle size, the 1:13 ratio was selected for further investigation.

### 3.2. Physicochemical Properties of THY@β-CD Microcapsules

**Figure 1:**
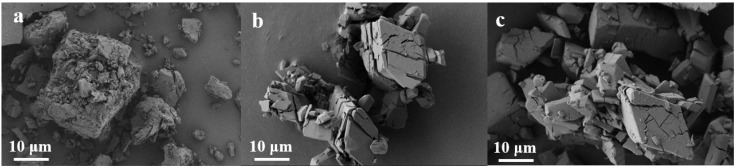
SEM images of β-CD (**a**), THY (**b**), THY@β-CD (**c**).

Figure 1a revealed the inherent tubular structure of β-CD, affirming its suitability as a microencapsulation wall material [35], with particles presenting as irregular masses featuring rough surface folds. In contrast, native THY displayed a highly crystalline, smooth morphology (Figure 1b). Figure 1c confirmed successful encapsulation, showing THY crystals incorporated within β-CD.

**Figure 2:**
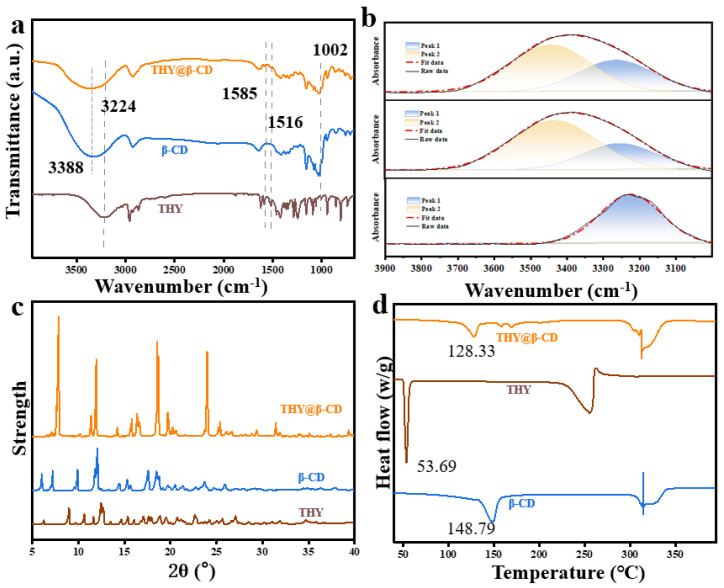
Characterization of β-CD, THY, and THY@β-CD: FTIR spectra (**a**); deconvoluted FTIR spectra (**b**); XRD patterns (**c**); DSC thermograms (**d**).

FTIR analysis (Figure 2a) provided molecular-level evidence supporting the inclusion mechanism. A distinct blueshift and narrowing of the O–H stretching vibration band were observed following complexation (Figure 2b). Deconvolution of this region further indicated a substantial decrease in the spectral contribution at ~3388 cm^−1^, which is associated with the self-assembled hydrogen-bonding network of β-CD, along with a pronounced increase in intensity at ~3224 cm^−1^. This shift in hydrogen-bonding populations confirms the disruption of native β-CD interactions and the establishment of specific host–guest hydrogen bonds, consistent with the incorporation of THY into the apolar cavity and its transition into an isolated microenvironment. Additionally, the characteristic aromatic C=C stretching vibration of THY at 1585 cm^−1^ was significantly attenuated, as were other vibrational bands between 1516 and 1002 cm^−1^, indicating restricted molecular mobility and successful encapsulation [36]. XRD patterns (Figure 2c) revealed a fundamental structural reorganization, evidenced by the disappearance of cage-type β-CD reflections and the emergence of new peaks consistent with a channel-type packing motif [37]. This architecture, with THY confined within linear hydrophobic channels, is expected to facilitate a more ordered diffusion pathway, thereby potentially influencing the release profile of the guest molecule [38]. TGA results (Figure 2d) confirmed enhanced thermal stability. The inclusion complex exhibited a two-stage decomposition process. The first stage, showing a significantly elevated onset temperature (128.33 °C) compared to pure THY (53.69 °C), demonstrates the protective effect of the β-CD matrix. This was corroborated by comparison with pure β-CD, which degrades at approximately 300 °C under identical conditions, affirming that the low-temperature mass loss corresponds to the release of stabilized and confined THY. A subsequent decomposition event at 312.51 °C, coinciding with the degradation of the β-CD host, confirmed the formation of a homogeneous complex.

Collectively, these findings affirm the successful formation of the THY@β-CD inclusion complex and support a coherent mechanism: hydrophobic interactions drive the encapsulation of THY into the apolar β-CD cavity, inducing a structural transition of the host into a channel-type framework that physically confines the guest. This synergy leads to enhanced thermal stability and provides a structural basis for modulated release properties [39].

### 3.3. Determination of the Sustained-Release Properties of THY@β-CD Microcapsules

**Figure 3:**
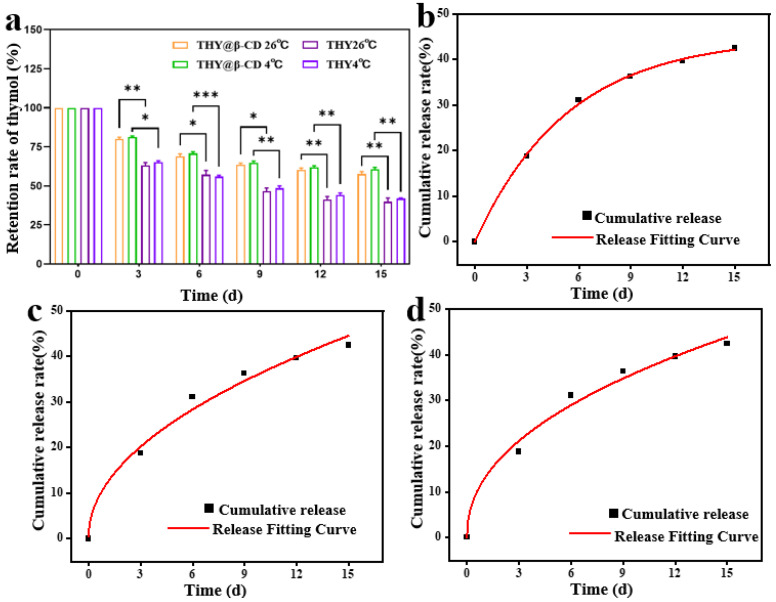
THY retention in THY@β-CD microcapsules versus free THY at 26 °C and 4 °C over 15-day storage (**a**). Data are presented as mean ± SD (*n* = 3). Significance between treatments at the same temperature was determined by repeated-measures two-way ANOVA with Bonferroni post hoc test (* *p* < 0.05, ** *p* < 0.01, *** *p* < 0.001). First-order (**b**), second-order (**c**), and third-order (**d**) release kinetics curves of THY@β-CD microcapsules.

The sustained-release characteristics of THY@β-CD microcapsules were characterized over a 15-day period under light-protected conditions using a repeated-measures ANOVA with between-subject factors of Treatment (microencapsulated vs. free THY) and Temperature (26 °C vs. 4 °C) with Bonferroni adjustment (Figure 3a). Analysis revealed a significant Treatment × Time interaction (*p* < 0.05). Post hoc tests indicated markedly greater THY retention in microcapsules than in free THY at both 26 °C (57.53 ± 1.17% vs. 39.91 ± 2.66%; ** *p* < 0.01) and 4 °C (62.18 ± 1.75% vs. 44.88 ± 1.43%; ** *p* < 0.01), with improved retention observed at the lower temperature. This enhancement is ascribed to the stabilizing influence of the β-CD inclusion complex, which shields the guest molecule from external factors [40]. A statistically significant main effect of temperature was also identified (*p* < 0.05). However, Bonferroni-corrected comparisons within each treatment group showed no significant release differences between 26 °C and 4 °C for either microencapsulated or free THY (*p* > 0.05), indicating that the influence of temperature was secondary to that of treatment. The significant Treatment × Time interaction further supports that microencapsulation substantially modified release kinetics over time relative to free THY. The consistently higher retention observed in microcapsules across temperature conditions, together with absent intra-treatment temperature effects, implies that host-guest complexation within the β-CD cavity mitigates the effect of thermal kinetic energy on release dynamics, thereby promoting stability under fluctuating temperature conditions [41].

Given its practical relevance as a storage temperature and its association with a more sustained release profile, the 4 °C condition was therefore selected for subsequent kinetic modeling to investigate the underlying release mechanisms. The release profile exhibited biphasic behavior: an initial rapid phase (0–6 days), governed by Fickian diffusion due to a high concentration gradient, was followed by a sustained release phase (6–15 days). Release data were fitted to conventional kinetic models (Table 4). Model selection was based on the AIC [42] and RMSE [43]. Among the models evaluated (Figure 3b–d), the first-order model was identified as optimal, demonstrating superior goodness-of-fit across all statistical metrics. It exhibited the highest coefficient of determination (R^2^ = 0.99877), the lowest residual sum of squares (RSS_(Residual Sum of Squares)_ = 0.83), the smallest RMSE (0.37%), and the most favorable AIC value (−6.76). The associated rate constant k was 44.89539 d^−1^. These results strongly suggest that the release of THY follows a diffusion-driven process, consistent with the release mechanisms observed in other essential oil/cyclodextrin systems where the release rate is proportional to the remaining core material within the microcapsules [44,45]. In contrast, both the Higuchi and Korsmeyer–Peppas models showed significantly lower goodness-of-fit, with reduced R^2^ values (<0.99), higher RSS and RMSE values, and elevated AIC scores. It is noteworthy that the release exponent n derived from the Korsmeyer–Peppas model was 0.45269—a value near the critical threshold between Fickian diffusion (n ≤ 0.45) and non-Fickian transport (0.45 < n < 0.89) as defined by the Korsmeyer–Peppas model [46]. This suggests that the release mechanism may not be purely Fickian and could involve minor contributions from other processes. Nevertheless, based on the objective statistical comparison of AIC and RMSE, the first-order model remains the most robust and reliable descriptor of the release kinetics over the entire period.

### 3.4. Antibacterial Properties of the Composite Coating

**Figure 4:**
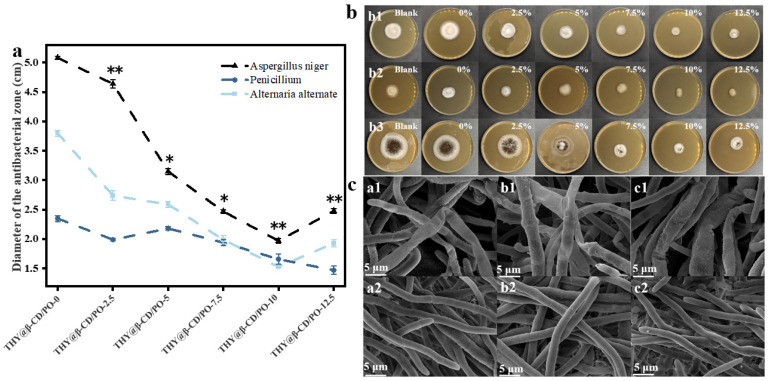
Inhibition zone diameters of THY@β-CD/PO coatings against *Alternaria alternate*, *Penicillium* sp., and *Aspergillus niger* at varied microcapsule loadings (wt%) (**a**). Significance between treatments was determined by repeated-measures two-way ANOVA with Bonferroni post hoc test (* *p* < 0.05, ** *p* < 0.01); visual antifungal effects (**b**) on *Alternaria alternate* (**b1**), *Penicillium* sp. (**b2**), and *Aspergillus niger* (**b3**); SEM images (**c**) of THY@β-CD/PO-10 coating on *Alternaria alternate* (**a1**,**a2**), *Penicillium* sp. (**b1**,**b2**), and *Aspergillus niger* (**c1**,**c2**).

Disk diffusion assays demonstrated a concentration-dependent antifungal effect of THY@β-CD/PO composite coatings against key postharvest pathogens, including *Alternaria alternate*, *Penicillium* sp., and *Aspergillus niger*. A two-way ANOVA revealed significant main effects of both microcapsule concentration and fungal species on colony diameter (*p* < 0.0001), along with a significant interaction between these factors (*p* < 0.0001). As illustrated in Figure 4a, colony diameters decreased with increasing microcapsule concentration. Post hoc Bonferroni’s test confirmed that all treatment groups (2.5–12.5 wt%) resulted in significantly smaller diameters compared to the blank control (0 wt%) (*p* < 0.05), with the 5, 10, and 12.5 wt% groups exhibiting stronger inhibition (** *p* < 0.01). Visual evidence of antifungal activity was provided in Figure 4b (* *p* < 0.05). The largest inhibition zones were observed at THY@β-CD/PO-12.5 wt% for *Penicillium* sp. (1.47 ± 0.07 cm), and at THY@β-CD/PO-10 for *Alternaria alternate* (1.53 ± 0.03 cm) and *Aspergillus niger* (1.97 ± 0.04 cm). A decline in activity beyond 10 wt% was ascribed to microcapsule aggregation [47]. In contrast, blank PO coatings enhanced fungal growth, likely due to nutritive components from starch and the lack of antimicrobial properties in glycerol.

Further supporting these findings, time-dependent colony growth assays (Figure S2) indicated pronounced inhibition by both THY and THY@β-CD/PO-10 over a 5-day period. By day 5, inhibition of *Alternaria alternate* and *Aspergillus niger* was highly significant (**** *p* < 0.0001), and that of *Penicillium* sp. was also strongly significant (*** *p* < 0.001), underscoring the sustained efficacy of the microencapsulated formulation. SEM imaging (Figure 4c) corroborated these results, revealing structural anomalies in treated hyphae—such as deformation, shrinkage, and cytoplasmic leakage—consistent with disruption of cell wall integrity [48]. The controlled release profile and prolonged antimicrobial action of THY@β-CD microcapsules underscore their potential as effective agents for food preservation.

### 3.5. THY@β-CD/PO Composite Coating Characterization

**Figure 5:**
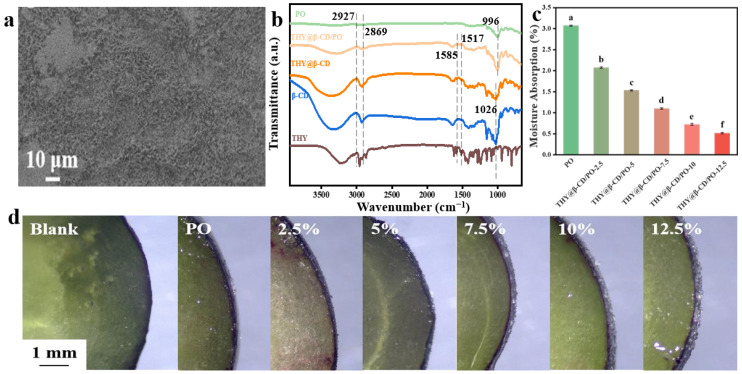
SEM image of blueberry surface after PO coating loaded with THY@β-CD microcapsules (**a**); FTIR spectrum of β-CD, THY, THY@β-CD, PO and THY@β-CD/PO (**b**). The mass increase of THY@β-CD/PO coating with different microcapsule dosages (**c**). Optical morphology of blueberry cut surface after coating with THY@β-CD/PO with different microcapsule dosages (**d**). The vertical lines indicate the standard deviation of the mean. Different superscript letters (a–f) indicate statistically significant differences (*p* < 0.05).

As shown in Figure 5a, the PO coating exhibited high loading capacity for THY@β-CD microcapsules, with uniform dispersion within the matrix enhancing the bacteriostatic activity of THY. This microstructural homogeneity was corroborated by Figure S1, which showed improved continuity and uniformity of coating coverage on blueberry surfaces treated with the THY@β-CD/PO composite compared to uncoated fruit.

FTIR analysis (Figure 5b) identified characteristic peaks corresponding to β-CD (C–H stretching at 2927 cm^−1^) and THY@β-CD (2927, 2869, and 1585 cm^−1^) within the composite coating, confirming the successful incorporation of both components. A discernible blue shift (Δν = +12 cm^−1^, *p* < 0.05) was detected in the C=C stretching vibrations (observed at 1585 and 1517 cm^−1^) compared to free microcapsules, suggesting alterations in intermolecular interactions, such as the formation of hydrogen bonds [49]. The presence of the PO-specific band at 996 cm^−1^, which is associated with water vibrations in amorphous regions of starch [50], indicated that the structural integrity of the starch backbone was preserved throughout the compositing process. These spectroscopic findings correlate with moisture absorption results (Figure 5c), where increased microcapsule loading led to a significant reduction in equilibrium water uptake (*p* < 0.05). This behavior is attributed to the shielding of hydrophilic domains in the starch matrix by the hydrophobic cavities of β-CD, thereby inhibiting moisture adsorption and diffusion [20]. Optical microscopy (Figure 5d) further revealed that the coating formed a continuous and uniform layer (<10 μm) on the berry surface. Elevated microcapsule content resulted in increased surface roughness, with clearly identifiable microcapsules corroborating successful integration. The combined reduction in hygroscopicity and the formation of a continuous thin-film structure collectively contribute to suppressed environmental vapor absorption, suggesting a potential mitigation of moisture-induced quality degradation during fruit storage.

**Figure 6:**
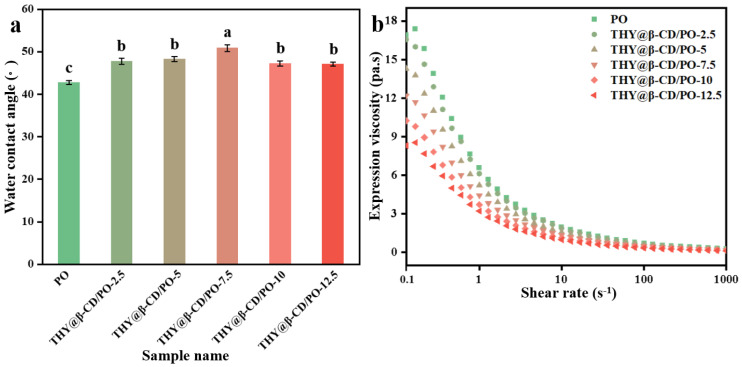
Water contact angle of THY@β-CD/PO coating with different microcapsule dosages (**a**); the flow behavior of THY@β-CD/PO coating with different microcapsule dosages (**b**); the vertical lines indicate the standard deviation of the mean. Different superscript letters (a–c) indicate statistically significant differences (*p* < 0.05).

The surface wettability of the composite coatings, which reflects resistance to liquid water spreading, was evaluated through water contact angle measurements (Figure 6a). In contrast to the reduced bulk hygroscopicity, the wettability was governed by the hydrophilic nature of the β-CD outer surface. The contact angle increased from 42.4° to 50.7° with higher THY@β-CD loading, yet remained below 90°, consistent with a hydrophilic interface. This behavior is attributed to two factors: (1) increased surface roughness induced by the microcapsules (Figure 5d), which amplifies intrinsic hydrophilicity as described by the Wenzel model [51]; and (2) exposure of hydroxyl groups on β-CD, providing hydrophilic sites. Although β-CD reduces bulk water uptake by shielding starch hydroxyls, its outer wall maintains moderate surface hydrophilicity. The combined influence of roughness and surface chemistry resulted in the observed non-monotonic trend in contact angle with increasing loading [52].

The viscosity of the coating solution, which reflects internal friction during flow and critically affects coating applicability, decreased with increasing shear rate (Figure 6b), indicating shear-thinning behavior and non-Newtonian characteristics even after microcapsule incorporation. Furthermore, apparent viscosity decreased with higher microcapsule loading, likely due to THY@β-CD intercalation between starch molecular chains. This reduces intermolecular interactions and disrupts chain packing, facilitating molecular sliding and enhancing fluidity [53].

Based on a comprehensive performance evaluation, THY@β-CD/PO-10 was selected for further study. At low shear (0.1 s^−1^), its viscosity (9.5 mPa·s) was 29.3% lower than that of THY@β-CD/PO-7.5 (12.3 mPa·s) and 47.2% lower than pure PO (18 mPa·s). Under high shear (10 s^−1^), the viscosity remained low (1.3 mPa·s, a 31.6% reduction compared to THY@β-CD/PO-7.5), indicating favorable storage stability and coating fluidity. Additionally, its hygroscopicity (0.8%) was 38.5% lower than that of THY@β-CD/PO-7.5 (1.3%, *p* < 0.01), consistent with FTIR evidence of hydrogen-bond alteration (Δν = +12 cm^−1^), confirming effective shielding of hydrophilic sites by β-CD. THY@β-CD/PO-10 also showed maximal inhibition of *Alternaria alternate* (1.53 ± 0.03 cm) and *Aspergillus niger* (1.97 ± 0.04 cm). While inhibition of *Penicillium* sp. (1.58 ± 0.06 cm) did not differ significantly from that of THY@β-CD/PO-12.5 (1.47 ± 0.07 cm, *p* > 0.05), it avoided the efficacy reduction observed at higher loading (1.82 ± 0.05 cm for *Penicillium* sp. with THY@β-CD/PO-12.5). Combined with optimized surface roughness and a contact angle of 47.2° ± 1.5° (Figure 5d and Figure 6a), THY@β-CD/PO-10 was identified as the optimal formulation for preservation trials.

### 3.6. Preservation Effects of THY@β-CD/PO Composite Coatings on Blueberries During Storage

**Figure 7:**
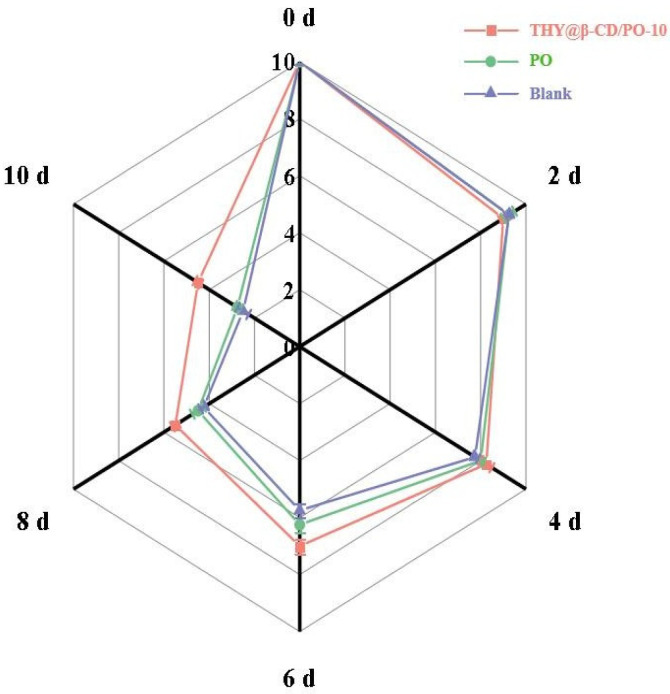
Sensory evaluation of blueberries treated with different methods during storage.

**Figure 8:**
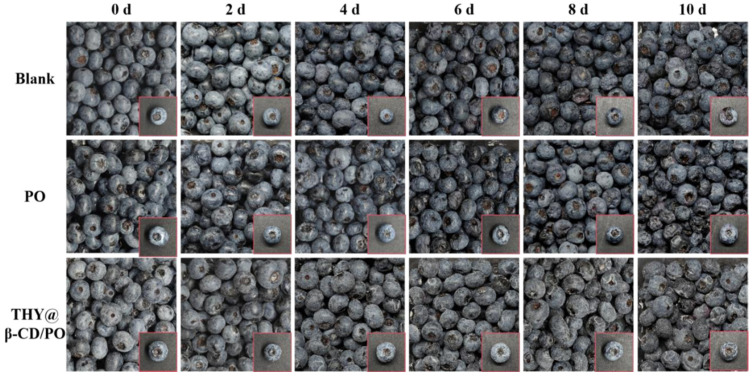
Apparent changes in blueberries treated with different methods during 10 days of storage.

Sensory evaluation of blueberries revealed distinct stage-dependent quality dynamics under different treatments (Figure 7). Initially, fruit coated with THY@β-CD/PO composite exhibited a slight reduction in sensory scores compared to the control during early storage (days 0–4: 9.0 ± 0.13 vs. 9.25 ± 0.25; *p* < 0.05), attributable to superficial textural heterogeneity and initial THY release [54]. As storage progressed, sustained antimicrobial release effectively suppressed microbial colonization, concomitantly delaying water loss and decay. By day 6, sensory scores of the composite-coated fruit significantly exceeded those of the PO-coated control (7.1 ± 0.5 vs. 5.8 ± 0.4; *p* < 0.01). Visual assessment (Figure 8) corroborated these results, with THY@β-CD/PO samples maintaining morphological integrity and surface gloss, in contrast to visible shriveling and coating fragmentation in controls. Color stability metrics further supported the preservation efficacy of the composite coating, as evidenced by minimal ΔE variation and maintained L* values (Table S1), indicating inhibited anthocyanin degradation. By day 8, treated fruit exhibited only mild shrinkage, whereas controls developed pronounced mold or softening. At endpoint storage (day 10), the THY@β-CD/PO coating significantly reduced overall quality loss by 47.3% relative to controls, a performance comparable to or exceeding other advanced coating systems [55].

**Figure 9:**
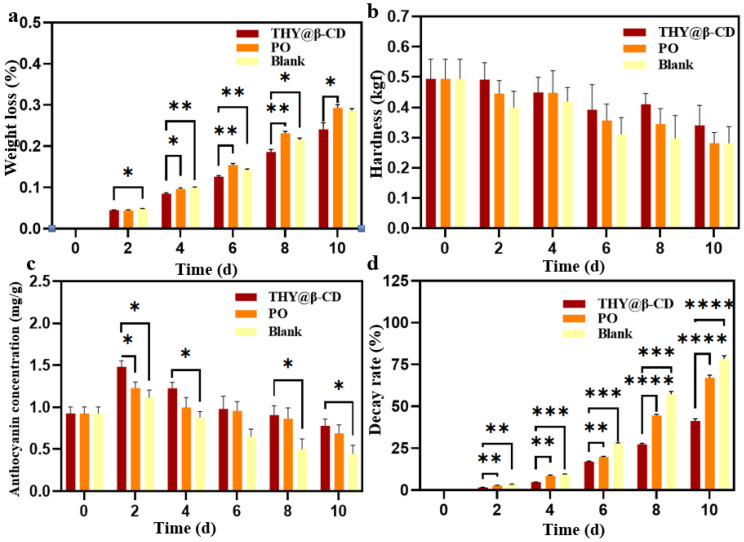
Effects of different treatments on the quality of blueberries during storage. Weight loss rate (**a**), hardness (**b**), anthocyanin content (**c**), decay rate. Treatments (**d**): untreated (control), PO, and THY@β-CD/PO-10 coating. Data are presented as mean ± standard deviation (*n* = 3). Statistical significance was determined by two-way ANOVA. Asterisks indicate significant differences between the THY@β-CD/PO-10 coating group and the untreated control group at each time point: * *p* < 0.05, ** *p* < 0.01, *** *p* < 0.001, **** *p* < 0.0001.

Figure 9 demonstrates a statistically significant interaction between treatment and time (*p* < 0.05) based on two-way ANOVA. In addition, both main effects—treatment and time—were highly significant (for time: *p* < 0.0001). As shown in Figure 9a (weight loss rate) and Figure 9b (hardness evolution), the THY@β-CD/PO composite coating group exhibited the least weight loss among all treatments during the initial 0–4 days of storage. Post hoc analysis of simple main effects (Figure 9a) revealed that the THY@β-CD/PO coating significantly reduced weight loss compared to the control group on day 2 (* *p* < 0.05), and outperformed both the PO and control groups by day 6 (** *p* < 0.01). Furthermore, a significant reduction relative to the PO group was maintained until day 10 (* *p* < 0.05). Although no statistically significant differences in hardness were detected among groups (Figure 9b), a consistent trend indicated improved hardness retention in the THY@β-CD/PO group, followed by the PO group, with the control showing the most pronounced decline. These findings indicate that the THY@β-CD/PO coating effectively mitigates weight loss and contributes to the maintenance of firmness in blueberries. In contrast, the PO group exhibited a marked increase in weight loss after day 6 (15.48 ± 0.67%). Previous studies have suggested that β-CD inclusion complexes act as protective barriers [56], and that the sustained release of THY may contribute to antimicrobial activity. The accelerated decay in control fruit was also associated with reduced surface moisture retention.

Anthocyanin content varied significantly across treatment groups during storage (Figure 9c). On day 2, the THY@β-CD/PO composite coating resulted in significantly higher anthocyanin levels (1.438 ± 0.07 mg/g) compared to the PO group (1.228 ± 0.07 mg/g; * *p* < 0.05) and the control group (1.113 ± 0.09 mg/g; * *p* < 0.05). This early increase may be attributed to suppressed respiration and the antioxidant activity of THY [57,58]. The THY@β-CD/PO coating also showed significantly higher anthocyanin retention than the PO group on day 4 (* *p* < 0.05), and compared to the control on days 8 and 10 (* *p* < 0.05). Overall, the composite coating effectively delayed anthocyanin degradation, demonstrating superior stabilization of metabolites throughout the storage period.

Antimicrobial efficacy exhibited time-dependent activity through sustained release (Figure 9d). On day 2, the THY@β-CD/PO composite coating showed significantly lower spoilage rates compared to both the control group (** *p* < 0.01) and the PO group (** *p* < 0.01). This difference was further amplified by day 6, with significantly enhanced antimicrobial performance relative to the PO group (*** *p* < 0.001). By day 10, the composite coating achieved a spoilage rate of 41.3 ± 2.94%, which was significantly lower than both the PO group (**** *p* < 0.0001) and the control group (78.5 ± 3.12%; **** *p* < 0.0001). The preservation mechanism integrates: (1) β-CD-mediated supramolecular encapsulation prolonging THY efficacy, (2) starch-based barrier functionality, and (3) time-dependent antimicrobial action [59]. Statistical modeling confirmed a shelf-life extension exceeding 10 days, establishing a theoretical basis for advanced preservation systems.

## 4. Conclusions

This study developed a novel THY@β-CD/PO composite coating that significantly enhances blueberry preservation. The core innovation involves microencapsulating THY within β-CD—at an optimal wall-to-core ratio of 13:1, yielding 73.24% encapsulation efficiency—to achieve sustained release via Fickian diffusion kinetics (R^2^ = 0.998), thereby overcoming the volatility and uncontrolled release limitations associated with direct THY incorporation. A 10 wt% loading of microcapsules into the PO matrix produced a transparent, hydrophilic coating that effectively reduced decay incidence by 47.3%, preserved 31% of firmness, and lowered weight loss by 33.4% relative to uncoated fruit over 10 days. Nevertheless, limitations persist, including a broad particle size distribution (PDI 0.68–0.73), which may affect release homogeneity and storage stability, as well as challenges related to production cost, scalability, and generalizability to other fruit varieties. Future research should aim to improve microcapsule uniformity, evaluate economic feasibility and scaling potential, and assess the environmental impact of coating residues.

## Figures and Tables

**Table 1:** The models of release behavior and parameters of fitted curves.

Model	Formula
first-order	Q = k × (1 − e^nt^)
Higuchi	Q = k × t^0.5^ + n
Korsemeyer–Peppas	Q = k × t^n^

where Q is the cumulative release rate; k is the release rate constant; n is the release mechanism parameter; t (day) is the storage time.

**Table 2:** Sensory and quality evaluation of blueberry.

Score	Flavor and Aroma	Appearance	Flesh Texture
8–10	Fruits are aromatic and have no other odor.	Fruit full, glossy and free from spoilage	Fruit firm, whole and elastic
6–8	Slight THY odor, no other off-flavors	Good shape, no spoilage	Fruit elastic, slightly less so than when picked
4–6	Strong musk vanillin odor, no other off-flavors	Fruits dull in color with a few scattered spots.	Fruit slightly soft
2–4	Flavors of wine and muscovado.	Less water loss, with a small amount of rotting	Fruit soft, little cracked
0–2	Strong THY flavor, musty and fermented, unacceptable gas	Loss of water and crumpling, severe rotting	Fruit soft, sticky texture, unformed

**Table 3:** Effect of wall to core ratio on microcapsule encapsulation efficiency, particle size, Zeta potential, and polydispersity index (PDI).

Core Wall Ratio (β-CD:THY)	Embedding Rate (%)	Particle Size (nm)	Zeta Potential (mV)	PDI
1:11	66.45 ± 1.21 ^d^	2120.79 ± 32.73 ^b^	−13.75 ± 0.15 ^a^	0.687 ± 0.021 ^ab^
1:12	68.48 ± 0.81 ^c^	2053.63 ± 29.54 ^c^	−14.32 ± 0.09 ^b^	0.706 ± 0.035 ^ab^
1:13	73.24 ± 0.82 ^a^	1927.77 ± 25.39 ^d^	−14.42 ± 0.12 ^c^	0.672 ± 0.028 ^b^
1:14	71.47 ± 0.78 ^b^	2017.62 ± 33.31 ^c^	−15.77 ± 0.1 ^c^	0.732 ± 0.027 ^a^
1:15	66.73 ± 0.65 ^d^	2184.34 ± 21.13 ^a^	−17.98 ± 0.12 ^d^	0.685 ± 0.032 ^ab^

Values are presented as means ± SD (*n* = 3). Mean values followed by different superscript letters (a–d) within the same column differ significantly (*p* < 0.05).

**Table 4:** Release kinetics models and fitted parameters including RSS, RMSE, and AIC.

Model	k	n	R^2^	RSS	RMSE	AIC
first-order	44.89539	0.1869	0.99877	0.83	0.37	−6.76
Higuchi	11.37336	0.45269	0.98592	6.76	1.06	12.40
Korsmeyer–Peppas	12.87733	0.45269	0.98350	7.91	1.15	13.48

## Data Availability

The original contributions presented in this study are included in the article/Supplementary Material. Further inquiries can be directed to the corresponding authors.

## Supplementary Materials

The following supporting information can be downloaded at: https://www.mdpi.com/article/10.3390/foods14173132/s1, Figure S1: SEM images of blueberry surface (a), PO coating (b), THY@ β- CD/PO coating (c). Figure S2: Colony growth diameters (cm) and photographs of Alternaria alternate treated with THY and THY@β-CD/PO-10 for 1 d, 3 d, and 5 d (a); colony growth diameters (cm) and photographs of Penicillium sp. treated with THY and THY@β-CD/PO-10 for 1 d, 3 d, and 5 d (b); colony growth diameters (cm) and photographs of Aspergillus niger treated with THY and THY@β-CD/PO-10 for 1 d, 3 d, and 5 d (c). Significance between treatments was determined by repeated-measures two-way ANOVA with Bonferroni post hoc test (*** *p* < 0.001, **** *p* < 0.0001); Table S1: L, a, b, and ΔE values of blueberries treated with different methods during storage.
