# Supplementary material for: β-Cyclodextrin/Thymol Microcapsule-Embedded Starch Coatings for Synchronized Antimicrobial Release and Shelf-Life Extension in Blueberries
Source: Foods. 2025 Sep 7;14(17):3132. doi: 10.3390/foods14173132 (PMC12428724; doi:10.3390/foods14173132)
Supplement: Supplementary file 1 [file foods-14-03132-s001.zip › foods-3820885-supplementary.pdf]

β-Cyclodextrin/Thymol Microcapsule-Embedded Starch Coatings for Synchronized Antimicrobial Release and Shelf-Life Extension in Blueberries

Xiangyue Li [1], Yuxin Liu [1], Jiayi Zheng [1], Xiaoyi Zhu [1], Weirui Fang [1], Shanshan Lei [1], Weiran Zhuang [1], Jing Wu [1], Tong Hao [1], Sulin You [1], Xi Wei [1], Wen Qin [1], Yaowen Liu [1,*], Mingrui Chen [1,*]

[1] College of Food Science, Sichuan Agricultural University, Yaan, China
* Correspondence: ; yaowenliu@sicau.edu.cn (Y. Liu); mingrui.chen@sicau.edu.cn (M. Chen)

Supplemental Materials

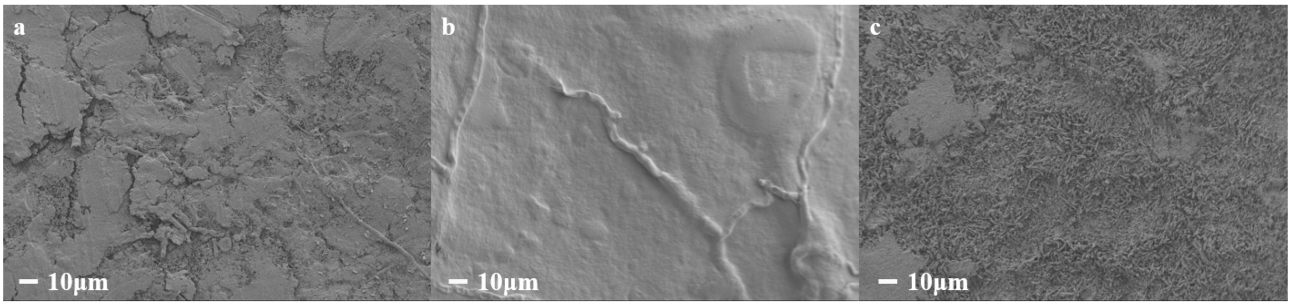

Figure S1. SEM images of blueberry surface (a), PO coating (b), THY@ β- CD/PO coating (c).

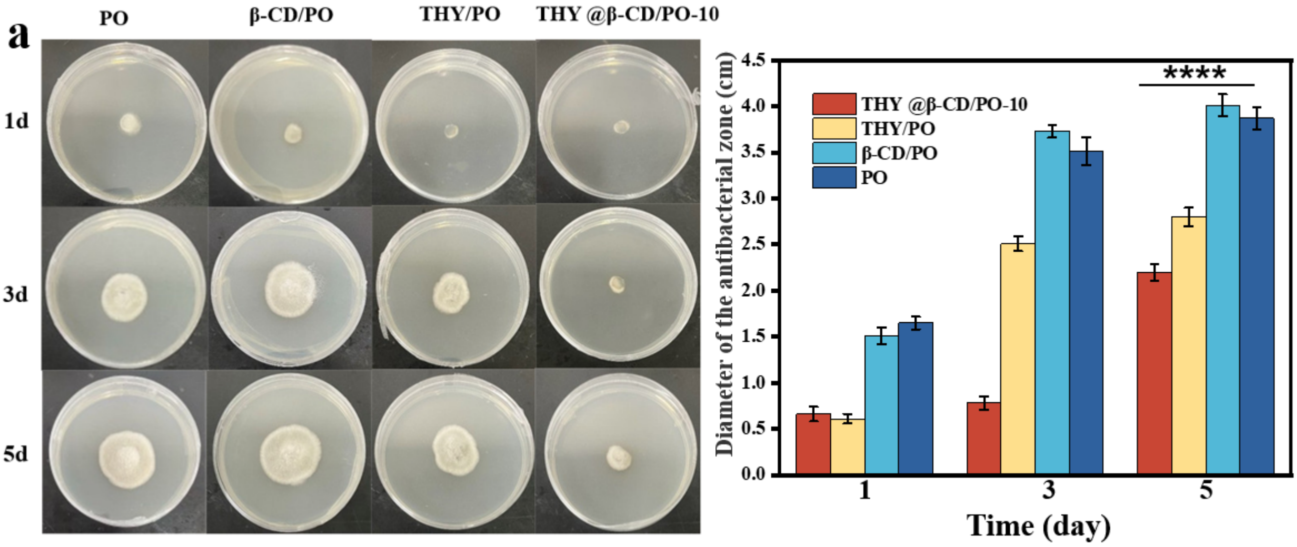

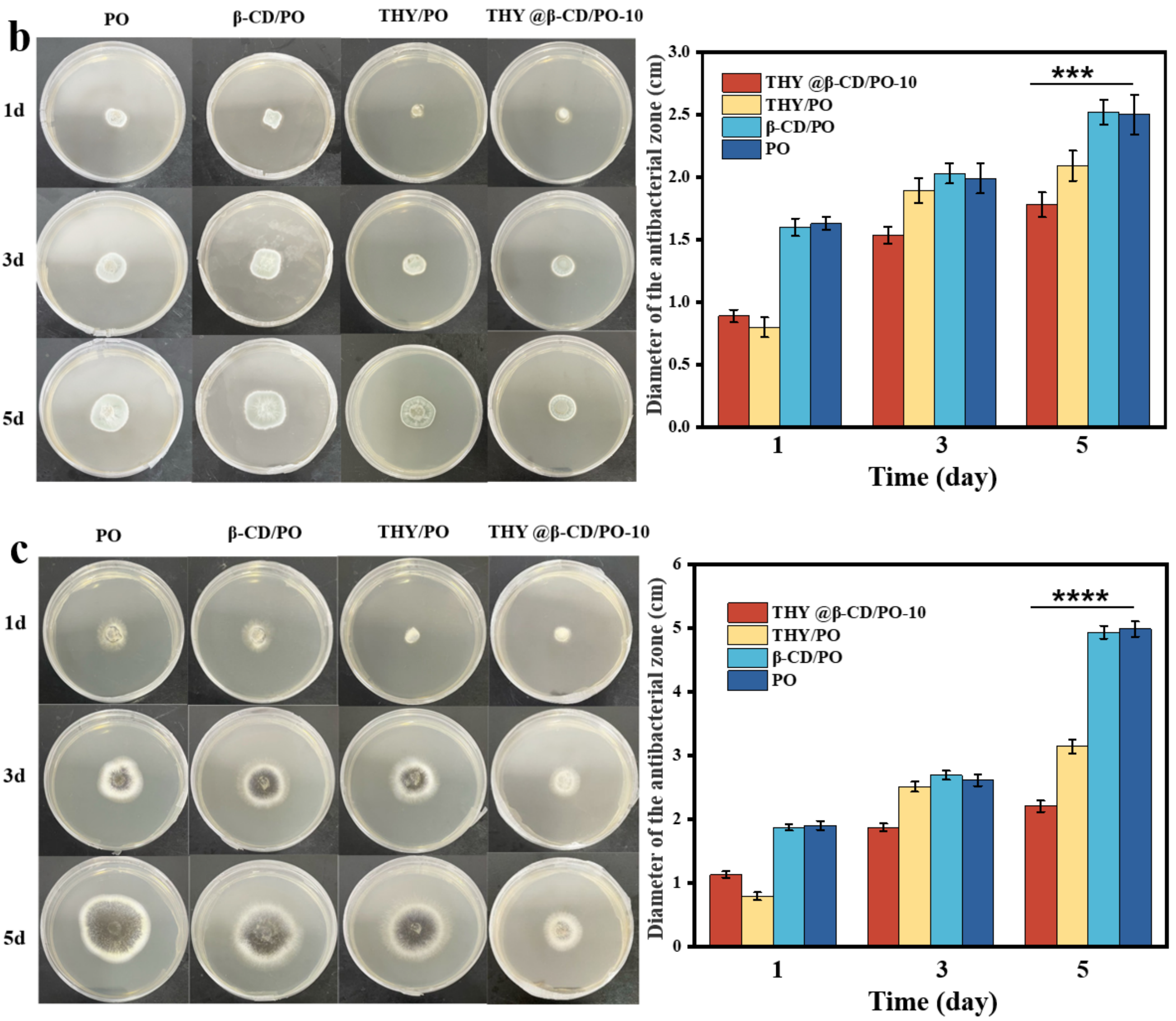

Figure S2. Colony growth diameters (cm) and photographs of *Alternaria alternate* treated with THY and THY@β-CD/PO-10 for 1 d, 3 d, and 5 d (a); colony growth diameters (cm) and photographs of *Penicillium sp.* treated with THY and THY@β-CD/PO-10 for 1 d, 3 d, and 5 d (b); colony growth diameters (cm) and photographs of *Aspergillus niger* treated with THY and THY@β-CD /PO-10 for 1 d, 3 d, and 5 d (c). Significance between treatments was determined by repeated-measures two-way ANOVA with Bonferroni post hoc test (****$p < 0.001$*, *****$p < 0.0001$*).

Table S1. L, a, b, and ΔE values of blueberries treated with different methods during storage.

Day	Groups	L*	a*	b*	ΔE
0	THY@β-CD/PO-10	48.44±1.46^{a}	-1.28±0.12^{a}	-2.78±0.71^{a}	38.07±1.48^{a}
	PO	46.60±1.91^{a}	-1.24±0.19^{a}	-2.92±0.85^{a}	39.93±1.79^{a}
	Blank	48.00±6.05^{a}	-1.36±0.28^{a}	-3.26±0.95^{a}	38.60±5.87^{a}

	THY@β-CD/PO-10	47.09±1.26^{a}	-1.17±0.09^{ab}	-2.29±0.83^{a}	39.60±1.23^{b}
2	PO	43.51±3.36^{a}	-0.94±0.24^{b}	-2.55±1.17^{a}	43.20±3.25^{a}
	Blank	44.15±2.62^{a}	-1.32±0.16^{a}	-2.68±0.66^{a}	42.59±2.55^{ab}
	THY@β-CD/PO-10	48.58±3.22^{a}	-0.95±0.15^{b}	-2.00±0.56^{a}	41.72±3.15^{a}
4	PO	49.34±3.08^{a}	-1.36±0.08^{a}	-2.14±3.05^{a}	41.31±2.99^{a}
	Blank	50.76±4.17^{a}	-1.38±0.19^{a}	-3.67±0.23^{a}	39.69±4.08^{a}
	THY@β-CD/PO-10	49.58±2.47^{a}	-0.94±0.09^{b}	-2.19±0.46^{b}	37.64±2.45^{a}
6	PO	48.83±2.50^{a}	-1.48±0.28^{a}	-3.69±0.42^{a}	38.59±2.47^{a}
	Blank	46.36±6.82^{a}	-1.17±0.42^{ab}	-3.12±1.12^{ab}	40.99±6.63^{a}
	THY@β-CD/PO-10	48.57±2.90^{a}	-0.88±0.23^{a}	-2.15±0.56^{a}	41.55±2.82^{a}
8	PO	43.83±6.25^{a}	-0.43±0.71^{a}	-2.23±1.65^{a}	46.31±6.06^{a}
	Blank	44.44±1.09^{a}	-1.04±0.17^{a}	-3.31±0.35^{a}	45.76±1.06^{a}
	THY@β-CD/PO-10	50.50±0.67^{a}	-0.84±0.20^{a}	-2.75±0.38^{b}	36.94±0.64^{b}
10	PO	44.36±0.88^{b}	-0.53±0.59^{a}	-2.96±0.26^{ab}	43.07±0.87^{a}
	Blank	43.46±1.24^{b}	-0.85±0.08^{a}	-3.19±0.18^{a}	43.97±1.22^{a}

Values are presented as means ± SD (n=3). Values marked with different superscript letters (a, b) within the same column differ significantly ($p < 0.05$).
